# Genetic Diversity and Population Structure of *Coilia nasus* Revealed by 2b-RAD Sequencing

**DOI:** 10.3390/biology12040600

**Published:** 2023-04-14

**Authors:** Shuangmeng Zhang, Zisheng Xu, Lifei Luo, Shuxin Gu, Zhen Hu, Shiming Wan, Zexia Gao

**Affiliations:** 1 College of Fisheries, Huazhong Agricultural University, Wuhan 430070, China; 2 Hubei Hongshan Laboratory, Wuhan 430070, China; 3 Zhenjiang Jiangzhiyuan Fishery Technology Co., Ltd., Zhenjiang 212213, China; 4 Hubei Provincial Aquatic Technology Promotion Station, Wuhan 430060, China

**Keywords:** genetic diversity, genetic structure, gene flow, *Coilia nasus*, 2b-RAD sequencing

## Abstract

**Simple Summary:**

*Coilia nasus* is a migratory fish species with high nutritional and economic value, and naturally inhabits the middle and lower reaches of the Yangtze River and offshore China. Since the 1970s, the continuous deterioration of the habitat environment and continuous high-intensity fishing have led to a sharp decline in the germplasm resources of the *Coilia nasus*, which has been listed in the Red List of Threatened Species by the International Union for Conservation of Nature. Artificial farming has gradually become an important way to protect and utilize the germplasm resources of *Coilia nasus*, but limited germplasm sources and unscientific breeding strategies have put the germplasm resources and genetic diversity of *Coilia nasus* at risk, which limits its resources protection and utilization. The aim of this study was to assess the germplasm resources of *Coilia nasus* by analyzing the genetic diversity and genetic structure of its natural and farmed populations. In conclusion, the present study provides a reference for germplasm conservation and breeding strategy optimization in *Coilia nasus*, and contributes to the healthy development of *Coilia nasus* aquaculture.

**Abstract:**

*Coilia nasus* is a threatened migratory species in the Yangtze River Basin. To reveal the genetic diversity of natural and farmed populations of *C. nasus* and the status of germplasm resources in the Yangtze River, the genetic diversity and structure of two wild populations (Yezhi Lake: YZ; Poyang Lake: PY) and two farmed populations (Zhenjiang: ZJ; Wuhan: WH) of *C. nasus* were analyzed using 44,718 SNPs obtained via 2b-RAD sequencing. The results indicate that both the wild and farmed populations had low genetic diversity, and germplasm resources have undergone varying degrees of degradation. Population genetic structure analyses indicated that the four populations may have come from two ancestral groups. Different amounts of gene flow were identified among WH, ZJ, and PY populations, but gene flow among YZ and other populations was low. It is speculated that the river–lake isolation of Yezhi Lake is the main cause of this phenomenon. In conclusion, this study revealed that genetic diversity reduction and germplasm resource degradation had occurred in both wild and farmed *C. nasus*, suggesting that conservation of its resources is of great urgency. This study provides a theoretical basis for the conservation and rational exploitation of germplasm resources for *C. nasus.*

## 1. Introduction

*Coilia nasus* is a migratory fish species with high nutritional and economic value and naturally inhabits the middle and lower reaches of the Yangtze River and offshore China [1,2]. *C. nasus* was once abundant and one of the most important fish resources in the Yangtze River [3]. However, changes in hydrological conditions, such as river–lake isolation and water quality deterioration, have led to *C. nasus* habitat destruction since the 1970s. Meanwhile, continuous high-intensity fishing has also led to a sharp drop in its catch, from 3750 tons in 1973 to 12 tons in 2012. Its reproductive rate and body size have also declined [3,4,5]. Accordingly, *C. nasus* has been listed on the IUCN Red List of Threatened Species [6,7]. Recently, with the implementation of the national policies of the Yangtze River Protection Law and the Ten-Year Fishing Ban in the Yangtze River, the wild catch of *C. nasus* is prohibited and the consumption demand for *C. nasus* is mainly satisfied by farming. The artificial breeding and culture of *C. nasus* have gradually developed [8]. However, assessment of the wild and farmed population structure and diversity is limited. Therefore, it is necessary to explore the population genetic characteristics of *C. nasus* to provide data references for its conservation and resource utilization [9].

Population genetic diversity is the basis of biodiversity and also a measure of the evolutionary potential of species. Understanding populations’ genetic structure is beneficial to developing targeted conservation measures and realizing species’ recovery potential [10]. Genetic diversity research has provided important theoretical guidance for resource assessment and conservation in various species, including mammals, birds, and plants [11,12,13]. In fish, genetic diversity analysis also provides important data for the conservation and rational utilization of fish germplasm resources and the healthy development of aquaculture [10,14,15]. Zhai et al. (2019) analyzed the genetic diversity and population structure of the endemic carp *Ancherythroculter nigrocauda* in the upper Yangtze River utilizing the mitochondrial cytochrome b gene and SSRs (simple sequence repeats). The genomic polymorphism and genetic diversity were revealed using a panel of single-nucleotide polymorphisms (SNPs) in wild and captive populations of *Arapaima gigas* through ddRAD sequencing [15]. In *C. nasus*, existing studies have mainly focused on its species identification, development, and migratory adaptation mechanism [16,17,18]. Studies on genetic diversity and genetic structure have been carried out based on mitochondrial genes, AFLP (amplified fragment length polymorphism), and SSR markers. Han et al. (2015) used AFLP markers to analyze the genetic structure between *C. nasus* from the East China Sea and the Yellow Sea, revealing the coastal dispersal pattern in this estuarine species [19]. Yu et al. (2019) revealed that the genetic diversity of artificially selected *C. nasus* populations was lower than that of the wild populations in the Yangtze River based on SSR markers [9]. Population genetic structure analysis of *C. nasus* by mitochondrial Cyt b showed low genetic differentiation between different river sections of the Yangtze River [20]. However, to date, few studies have explored the genetic structure of wild and farmed *C. nasus* populations and assessed their genetic diversity based on genome-wide variation information.

The rapid development of high-throughput sequencing technology provides an efficient way of obtaining genome variants for population genetics studies. As one of the most effective methods for single-nucleotide polymorphism (SNP) identification, restriction-site associated DNA (RAD) sequencing has been widely used in population genetic studies of aquatic animals. The 2b-RAD technology has the advantage of simplicity and flexibility as a means of providing genome-wide genotyping information [21]. For instance, based on 2b-RAD sequencing, the low genetic diversity of five golden-backed carp (*Cyprinus carpio* var. *Jinbei*) populations in Guizhou, China, was revealed [22]. Based on RAD sequencing, significant genetic differentiation of *Monopterus albus* in different regions of China was identified [23]. With the rapid growth of *C. nasus* farming, limited germplasm sources and unscientific breeding strategies could occur and result in a decline in the germplasm and genetic diversity of *C. nasus* in the farmed populations. At present, the genetic diversity and population genetic structure of the natural and farmed populations of *C. nasus* are still not fully understood. Therefore, it is necessary to assess the genetic diversity and population structure of *C. nasus* in the farmed populations and compare them with those of wild populations.

The goal of this study was to analyze the genetic diversity and population structure and assess the status of germplasm resources for two wild and two farmed populations of *C. nasus* based on genome-wide SNP discovery using 2b-RAD sequencing technology. The results of this study are of great significance for the protection and scientific utilization of the *C. nasus* germplasm and breeding program.

## 2. Materials and Methods

### 2.1. Ethics Statement

This study was conducted in accordance with the “Guidelines for Experimental Animals” of the Ministry of Science and Technology (Beijing, China). The protocols for fish handling and sampling were approved by the Institutional Animal Care and Use Ethics Committee of Huazhong Agricultural University (No. HZAUFI-2023-0009).

### 2.2. Sample Collection and DNA Extraction

**Figure 1:**
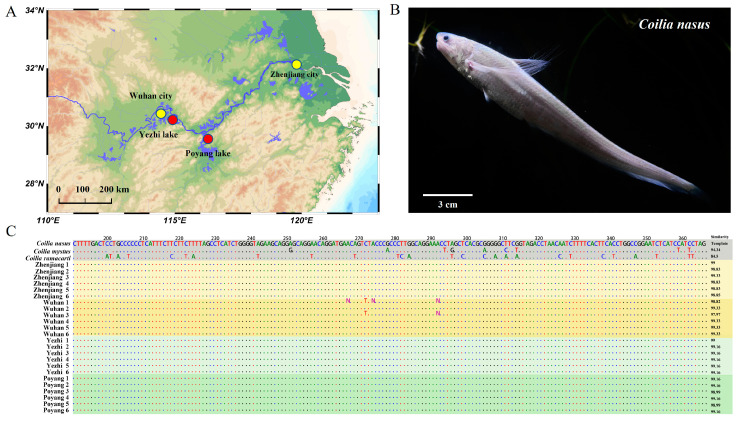
Sample collection and identification of *Coilia nasus*. (**A**) Geographical location of sampling sites. The red dots indicate the wild populations and the yellow dots indicate the farmed populations; (**B**) appearance characteristics of *Coilia nasus*; (**C**) visualization of partial sequence alignment results. The gray background indicates the published COI sequences of *Coilia nasus*, *Coilia mystus*, and *Coilia brachygnathus* in the database, and the differently colored backgrounds indicate different wild and farmed populations.

Two wild populations of *C. nasus* were sampled from Yezhi Lake (YZ) in Hubei Province (*n* = 18) and Poyang Lake (PY) in Jiangxi Province (*n* = 6), and two farmed populations (wild type and farmed type, respectively, as defined by FAO) were sampled from Zhenjiang (ZJ) in Jiangsu Province (*n* = 40) and Wuhan (WH) in Hubei Province (*n* = 30). (Figure 1A,B). It was stated that all farmed fish originated from ancestor parents caught from the Yangtze River. For all 94 individuals, a piece of fin was removed and stored in anhydrous ethanol at −20 °C. The genomic DNA was extracted using the ammonium acetate method [24] and the DNA quality was analyzed using nanodrop spectrophotometric and agarose gel electrophoresis. The high-quality DNA samples were then stored at −20 °C for further use.

### 2.3. Species Identification

All fish individuals in this study were confirmed as *C. nasus* via sequencing of the mitochondrial COI gene. The DNA of each fish was amplified as the template using specific COI primers for *C. nasus* (COI-F: TATTTAGTATTCGGTGCCTG; COI-R: TGCTACTTCTCGTTTGGC) [25]. The PCR products were detected via agarose gel electrophoresis and sequenced using Wuhan Tsingke Biotechnology. The COI sequences generated from each individual were aligned with the published COI sequences of *C. nasus*, *C. mystus*, and *C. brachygnathus* in the Barcode of Life Data System database (http://www.boldsystems.org (accessed on 1 May 2022)). The alignment results were visualized using MEGA X (version 10.2.6; www.megasoftware.net (accessed on 1 December 2022)). The accession number of *C. nasus* from BOLD is AAF3963.

### 2.4. 2b-RAD Library Preparation and Sequencing

The libraries were constructed using 2b-RAD five-label tandem technology according to existing study [26]. Briefly, the genomic DNA was digested by the type IIB restriction enzyme BsaXI, and the cleavage products were ligated with five different sets of adaptors by T4 DNA ligase. According to the manufacturer’s instructions, the ligated products were amplified via PCR after linkage reaction, in which the five tags were sequentially linked in tandem according to the information of the five adaptors, and then the ligated products were enriched and purified using MinElute PCR purification kits. After SapI digestion and gel purification, the barcode sequences were added to the purified product. Products with different barcode numbers were purified using a MinElute PCR Purification Kit and pooled for sequencing using the Illumina NovaSeq 6000 PE150 sequencing platform from Qingdao OE Biotech Co., Ltd (Qingdao, China).

### 2.5. SNP Discovery and Genotyping

Reads containing enzyme recognition sites were extracted from the sequencing data, and the original sequencing data were filtered according to certain conditions. Specifically, reads containing more than 8% of N bases and low-quality reads (the number of bases whose quality value is lower than Q30 exceeds 15% of the total number of bases in reads) were filtered. The paired clean reads were spliced using Pear software (Version 0.9.6) to extract the sequencing reads of the samples corresponding to the library [27], and the sequenced reads without enzyme recognition sites were deleted to generate the high-quality sequences. The remaining sequences were mapped to the *C. nasus* reference sequence (SRS2757052) using SOAP software [17,28], and finally all SNP markers were typed using the maximum likelihood method (ML). Then, the SNPs obtained from the four populations were further filtered to obtain high-quality SNPs, in which loci containing only one allele, loci with N genomic bases, loci with more than two SNPs in one tag, loci with two typing loci at the same position, loci with less than 80% of individuals being typable in all samples, and loci with an MAF less than 0.01 were excluded.

### 2.6. Calculation of Genetic Parameters

The fixation index (Fst) between populations was calculated using R package StAMPP (Version 1.6.3 [29], the degree of population genetic differentiation was defined as Fst < 0.05 indicating low genetic differentiation; 0.05 < Fst < 0.15 indicating moderate genetic differentiation; 0.15 < Fst < 0.25 indicating large genetic differentiation, and Fst > 0.25 indicating extremely large genetic differentiation [30]. The Hardy–Weinberg equilibrium *p*-value (HW-P) and the nucleotide diversity (Pi) were calculated using VCF tools software (Version 0.1.14) [31]. The expected heterozygosity (He), observed heterozygosity (Ho), and effective number of alleles (Ne) were calculated with reference to Ji et al. [22].

The Reynolds’ genetic distance (DR) and gene flow (Nm) between populations were estimated from the interpopulation Fst with the equations DR = −ln (l − Fst) and Nm = (l − Fst)/4Fst. The polymorphism information content (PIC) was calculated according to the formula from Botstein [32], and PIC < 0.25 was considered as a low degree of polymorphism, 0.25 < PIC < 0.5 as moderately polymorphic, and PIC > 0.5 as highly polymorphic.

### 2.7. Analysis of Population Genetic Structure

Genome-wide SNPs were filtered by PLINK software (version 1.9), and SNPs without close linkage were selected. Then, population structure analysis was performed according to K = 1 to K = 10 using ADMIXTURE (version 1.3.0) software, and 10 different seeds were selected for 10 replicate analyses. The optimal K was determined to be K = 2 according to CV (Cross-Validation Error). PCA analysis of the obtained SNP markers was performed using plink2 software (version: 2.0) to obtain the two most influential eigenvectors [33]. The evolutionary tree was constructed using the neighbor-joining method, and the distance matrix (p-distance) was calculated using treebest software [34] (Version: 1.9.2). The reliability of the evolutionary tree was checked by bootstrap (1000 repetitions).

### 2.8. Data Availability

The sequencing data for four *C. nasus* populations are available in the NCBI Sequence Read Archive under accession number PRJNA899812.

## 3. Results

### 3.1. Species Identification

We conducted species identification of all individuals from two wild populations and two farmed populations to ensure the correctness of collected samples. After observation, the morphological characters of all 94 fish were found to be consistent with C. nasus. Furthermore, we performed molecular identification of the fish. After specific primer amplification, the COI gene sequences were sequenced and compared with the published COI sequences of *C. nasus*, *C. mystus*, and *C. brachygnathus*. Eventually, all the 94 individuals used in this study were identified as *C. nasus.* The COI sequence alignment of all individuals also proved the correctness of the species identification. (Figure 1C).

### 3.2. SNP Discovery via 2b-RAD Sequencing

**Figure 2:**
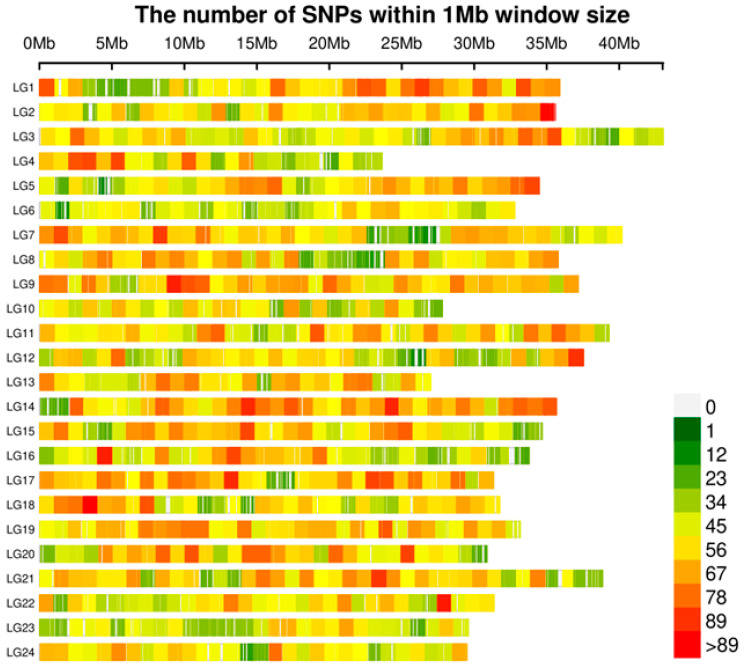
Distribution of SNPs on the different linkage groups of *Coilia nasus*. The color scale from green to red indicates the density of SNP distribution from low to high.

A range of 6,833,532 to 10,856,321 raw reads were obtained per sample. After filtering, 4,674,039 to 9,208,545 remained. The enzyme reads obtained from each sample were sequenced and aligned with the reference genome using SOAP software. A total of 44,718 SNPs were generated after quality filtering. Further analysis of the distribution of SNPs in the *C. nasus* genome indicated that the number of SNPs on linkage groups (LGs) ranged from 1247 (LG4) to 2353 (LG9) (Figure 2). In addition, the average distance between SNPs on linkage groups ranged from 15.478 kb/SNP (LG14) to 22.188 kb/SNP (LG23), with an average of 18.162 kb/SNP.

### 3.3. Genetic Diversity Analysis

The 44,718 generated SNPs were used for genetic diversity analysis. The *HW-P* values of the wild populations ranged from 0.7003 (ZJ) to 0.9257 (PY). The Hardy–Weinberg equilibrium test showed that all the HW-P values of farmed and wild populations were greater than 0.05, indicating that genetic equilibrium was maintained in all populations of *C. nasus* (Table 1). The mean observed heterozygosity (Ho) ranged from 0.0664 (YZ) to 0.1732 (WH), the mean expected heterozygosity (He) ranged from 0.0569 (YZ) to 0.1795 (WH), and the nucleotide diversity (Pi) ranged from 0.0588 (YZ) to 0.1830 (WH) (Table 1). The PIC values of all groups were less than 0.25, indicating that all populations had a low level of polymorphism. The number of effective alleles (Ne) ranged from 1.0949(YZ) to 1.2860 (WH). The lower genetic diversity of wild populations (YZ, PY) and farmed populations (ZJ, WH) indicated that they both exhibited varying degrees of germplasm degradation.

### 3.4. Genetic Differentiation Analysis

**Figure 3:**
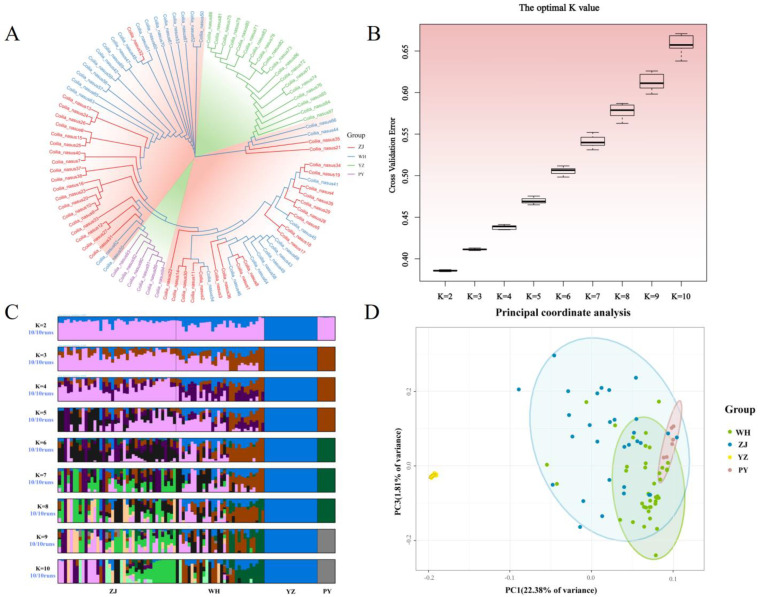
Population genetic structure analysis of four populations of *Coilia nasus* collected from farmed (WH and ZJ) and wild populations (PY and YZ). (**A**) Phylogenetic tree. The red background indicates the two breeding populations, ZJ and WH (farmed populations); the green background indicates the two wild populations, YZ and PY (wild populations). (**B**) *K*−value Cross-Validation Error box plot. (**C**) Pong clustering. *K*−value multiple repetition results are clustered, and 10/10 runs in the upper−left corner indicate that all 10 repetition results are the same. (**D**) Principal component analysis. Horizontal coordinates represent principal component; vertical coordinates represent principal component 3; each point indicates an individual, and different colors indicate different groups.

The neighbor-joining tree of all individuals was constructed based on SNPs obtained from 2b-RAD sequencing. The wild population YZ from Yezhi Lake clustered into a separate branch, indicating that it was genetically distant from other populations. The farmed populations ZJ and WH were more closely related, and their individuals were intermingled in one cluster and clustered in one branch with the wild population PY, which was clustered alone. Overall, the farmed populations ZJ and WH were more closely related to the wild group PY, while the wild group YZ was a separate genetic lineage (Figure 3A).

Further analysis of the genetic structure of the four *C. nasus* populations indicated that the optimal K-value was 2 (Figure 3B) according to CV (Cross-Validation Error), indicating that all the experimental individuals probably came from two common ancestors. The clustering analysis of each K-value using pong software also showed that the clustering result for K = 3 was the same as that for K = 2 and that ZJ and WH belonged to the same subgroup (Figure 3C). The results of the PCA analysis (Figure 3D) indicate that the two most influential eigenvectors, PCI and PC3, contributed 22.38% and 1.81%. The farmed populations ZJ and WH and the wild population PY were clustered into a branch., indicating that they most likely came from a common ancestor. In contrast, the wild population PY was distributed in a separate area, indicating that it is relatively distantly related to the other three populations.

The Fst of the four populations ranged from 0.0335 to 0.7518 (*p* < 0.001), and the DR ranged from 0.0341 to 1.3935 (Table 2). The two farmed populations, ZJ and WH, showed low differentiation (Fst < 0.05), while the wild population PY showed moderate differentiation from the two farmed populations (0.05 < Fst < 0.15), and the wild population YZ showed high differentiation from all other populations (Fst > 0.25). Meanwhile, in terms of the DR, the two farmed populations had the lowest DR of 0.0341. The wild population YZ had the largest DR of 1.3935 from the wild population PY. Compared with the PY population, the DR between the wild population YZ and the two farmed populations was higher.

### 3.5. Population Gene Flow Analysis

**Figure 4:**
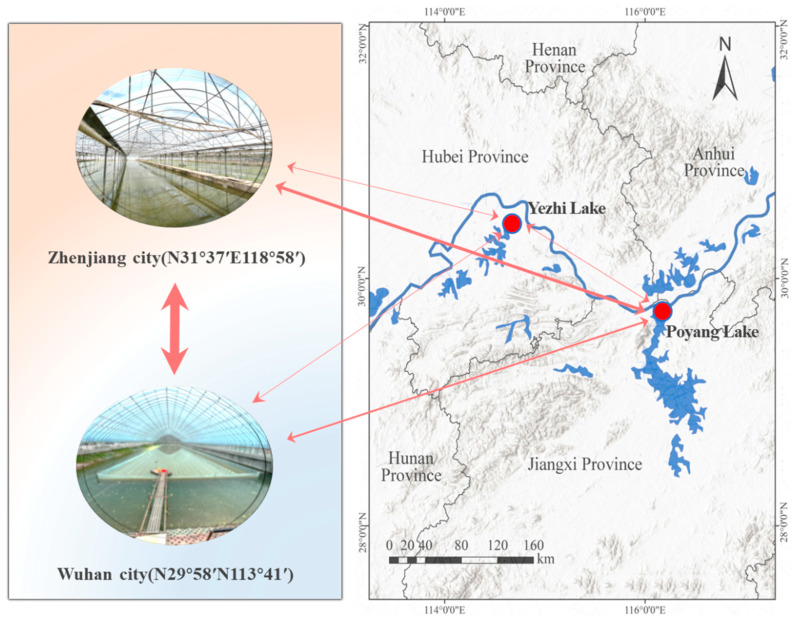
Schematic diagram of gene flow among the four *Coilia nasus* populations. The square on the left side represents the two breeding populations from Zhenjiang and Wuhan, and the red dots on the right map indicate the two wild populations from Yezhi Lake in Hubei Province and Poyang Lake in Jiangxi Province; the thickness of the arrow represents the size of gene flow between populations.

The Nm among the populations ranged from 0.1920 to 7.2127. The *Nm* between the farmed populations WH and ZJ was 7.2127, which was the highest level among the populations. The Nm between the wild population PY and the farmed populations ZJ and WH was 2.3678 and 1.6255, while the *Nm* between the wild population YZ and the farmed populations ZJ and WH was less than 1. These results indicate that the two farmed populations ZJ and WH had a higher level of gene exchange with the wild population PY, while the wild population YZ had little gene exchange with the other three populations. This was highly consistent with the results of the genetic structure analysis (Table 3, Figure 4)

## 4. Discussion

Population genetic diversity is the amount of variation observed among the DNA sequences of different individuals within a population, which is significant for the evolution of species survival, environmental adaptation, farming, and genetic breeding [35,36]. Populations with higher genetic diversity tend to have greater adaptive potential and richer genetic improvement properties, while low levels of genetic diversity are associated with a weak adaptive capacity, expression of harmful genes, deterioration of economic traits, and species degradation [20]. The study of genetic diversity can provide a useful basis for the development of strategies related to the conservation and rational use of natural resources [37]. The analysis of genetic diversity and structure among farmed and wild populations in this study provides important information for the conservation of the germplasm resources of *C. nasus* as well as the development of farming.

Important indicators of genetic diversity, including the mean heterozygosity and the polymorphism information content, are used to assess genetic diversity in populations, with higher values indicating higher genetic variation. In this study, based on Ho and He, the genetic diversity level of the four populations was low, and based on the PIC value (less than 0.25), the four populations showed low polymorphism. These results indicate that the wild and farmed populations in this study may have been degraded to varying degrees, and effective protection of wild germplasm and supplementation of farmed populations are urgently needed. Among them, the genetic diversity level of the wild population YZ was much lower than that of the other three populations. Considering the geographical features of the river–lake isolation of YZ Lake, it is speculated that the ancestral population of YZ may have been introduced from the Yangtze River through floods or other means [38]. The subsequent founder effect resulted in an extremely low level of genetic diversity in the YZ population [39]. The levels of genetic diversity in the two farmed populations were slightly higher than those in the two wild populations. The reason for this may be that the original parents of the farmed populations were collected from different reaches of the Yangtze River, and the offspring produced by random mating still had high genetic diversity. In general, the genetic diversity of wild and farmed populations was low, which is a departure from the results of genetic diversity studies of *C. nasus* in the Yangtze River system carried out more than ten years ago, in which the genetic diversity of *C. nasus* in the Yangtze River near Chongming and Changxing Island was high, and the germplasm resources were rich [40,41]. Changes in relevant indicators indicate a declining trend in the resources of wild populations of C. nasus over the past decade due to the impact of unreasonable fishing and changes in the ecological environment. Effective conservation and utilization of *C. nasus* germplasm resources for *C. nasus* are of the utmost importance.

The Fst and DR are essential parameters to measure the degree of genetic differentiation among populations [42]. In this study, the wild population YZ showed significant genetic differentiation from the other three populations, with the wild population PY showing moderate differentiation from the two farmed populations, while the two farmed populations showed a low degree of differentiation. The optimal number of subpopulations K was determined to be 2. Combined with the results of Fst, DR, and PCA analysis, we hypothesized that ZJ, WH, and PY were from the same ancestral population, while YZ was a separate lineage. In the case of gene flow (Nm), a value of more than 1 means that gene exchange between populations is more extensive and genetic differentiation is low, while a gene flow of less than 1 means that gene exchange between populations is blocked and genetic differentiation is increased [43]. In this study, the highest Nm was 7.3906 between the two farmed populations, which may be caused by the fact that both ancestral populations were from the Yangtze River. The Nm between the wild group PY and the farmed populations ZJ and WH was 2.5405 and 1.6820, respectively. As the largest freshwater lake in China, Poyang Lake is an important spawning and breeding habitat of *C. nasus*, and one of the few lakes connected to Yangtze River [44,45]. As a result of the implementation of the catch prohibition policy in the Yangtze River, more *C. nasus* can migrate to PY Lake to spawn and breed [46]. The ancestors of the farmed population of *C. nasus* were also caught from the Yangtze River, which may be the reason why the cultured populations are closely related to the PY populations. We also speculate that the extremely small *Nm* between the wild population YZ and other populations may be due to the geographic isolation of Yezhi Lake. Yezhi Lake was originally not connected to the Yangtze River, and the ancestors of *C.nasus* were probably brought into the Yezhi Lake by flooding. After multiple generations of reproduction and evolution, relatively independent genetic lineages formed. In addition, several individuals in the ZJ population deviated significantly from the main group and were genetically distant from other individuals. A similar situation was observed by Wang et al. (2021) when exploring the genetic diversity of nudibranchs, speculating that it results from missing samples of genetically related geographic populations [47]. Therefore, the ancestral populations of several individuals deviating from the ZJ population in PCA analysis may be distributed in adjacent waters, such as Taihu Lake and Dongting Lake, which were not collected. In the future, we need to collect different geographical populations of the *C.nasus* and conduct a more comprehensive assessment of its genetic diversity.

As one of the key species of Yangtze River, biological resource protection and the restoration of natural resources of *C. nasus* is very important. At the same time, it is of great significance to formulate scientific breeding and culture strategies for *C. nasus* to expand its production and meet its market demand. Accordingly, this study puts forward some suggestions to help the protection and utilization of the germplasm resources of *C. nasus*, specifically: establish protection areas for wild *C. nasus*, provide spawning grounds, bait grounds, and migration channels for *C. nasus*; analyze the genetic characteristics of different populations of the *C. nasus*, select parents with different genetic backgrounds for breeding to avoid germplasm degradation caused by inbreeding; meanwhile, scientifically carry out multiplication and release of the *C. nasus* population.

## 5. Conclusions

In conclusion, 2b-RAD sequencing technology was applied on *C. nasus* for genetic diversity evaluation. Genetic diversity reduction and germplasm resource degradation of wild and farmed *C. nasus* were revealed, suggesting that its resource conservation is of great urgency. The present study provides a reference for germplasm conservation and breeding strategy optimization in *C. nasus,* and contributes to the healthy development of *C. nasus* aquaculture.

## Figures and Tables

**Table 1:** Genetic diversity analysis of four populations of *Coilia nasus*.

Populations	Group	Ho	He	Pi	HW-P	PIC	Ne
Farmed	Zhenjiang (ZJ)	0.1514	0.1571	0.1593	0.7003	0.1334	1.2358
	Wuhan (WH)	0.1732	0.1795	0.1830	0.7517	0.1486	1.2860
Wild	Yezhi Lake (YZ)	0.0664	0.0569	0.0588	0.9244	0.0462	1.0949
	Poyang Lake (PY)	0.1190	0.1123	0.1249	0.9257	0.0920	1.1822

**Table 2:** The fixation index (Fst) (lower left) and genetic distance (DR) (upper right) values among four populations of *Coilia nasus*.

POP	ZJ	WH	YZ	PY
ZJ	-	0.0341	0.8339	0.1004
WH	0.0335	-	0.6323	0.1431
YZ	0.5656	0.4686	-	1.3935
PY	0.0955	0.1333	0.7518	-

**Table 3:** Population gene flow (Nm) among four populations of *Coilia nasus*.

POP	ZJ	WH	YZ	PY
ZJ	-			
WH	7.2127	-		
YZ	0.1920	0.2835	-	
PY	2.3678	1.6255	0.0825	-

## Data Availability

Publicly available datasets were analyzed in this study. This data can be found here: [PRJNA899812].
